# Continued decitabine/all-*trans* retinoic acid treatment: extended complete remission in an elderly AML patient with multi-hit *TP53* lesions and complex-monosomal karyotype

**DOI:** 10.1186/s13148-024-01737-4

**Published:** 2024-09-11

**Authors:** Johanna Thomas, Usama-Ur Rehman, Helena Bresser, Olga Grishina, Dietmar Pfeifer, Etienne Sollier, Konstanze Döhner, Christoph Plass, Heiko Becker, Claudia Schmoor, Maike de Wit, Michael Lübbert

**Affiliations:** 1 Department of Hematology, Oncology and Stem Cell Transplantation, Faculty of Medicine, University Medical Center Freiburg, Hugstetterstr. 55, 79106 Freiburg, Germany; 2 Faculty of Medicine, University Medical Center Freiburg, Clinical Trials Unit, Freiburg, Germany; 3 Cancer Epigenomics, German Cancer Research Center (DKFZ), Heidelberg, Germany; 4 Department of Internal Medicine III, University Hospital of Ulm, Ulm, Germany; 5 German Cancer Consortium (DKTK) and German Cancer Research Center (DKFZ), Partner Site Freiburg, Freiburg, Germany; 6 Department of Hematology, Oncology and Palliative Medicine, Vivantes Klinikum Neukoelln, Berlin, Germany

## Abstract

DNA-hypomethylating agents (HMAs) induce notable remission rates in AML/MDS patients with *TP53* mutations; however, secondary resistance often develops rapidly. In the DECIDER trial (NCT00867672), elderly AML patients (also those with adverse genetics) randomized to all-*trans* retinoic acid (ATRA) added to decitabine (DEC) attained significantly delayed time-to-resistance. An 82-year-old patient with a non-disruptive, in-frame *TP53* mutation (p.Cys238_Asn239delinsTyr, VAF 90%) and complex-monosomal karyotype attained a complete hematologic and cytogenetic remission with DEC + ATRA, with 3.7 years survival after 30 treatment cycles that were well-tolerated. Further HMA + ATRA studies appear warranted in AML/MDS patients of different genetic risk groups ineligible for more intensive treatment.

Trial registration: This trial was registered at ClinicalTrials.gov identifier: NCT00867672

## Introduction

With establishment of the DNA-hypomethylating agents (HMAs), azacitidine (AZA) and decitabine (DEC), there has been significant progress in treating older patients with acute myeloid leukemia (AML) ineligible for intensive chemotherapy. Adding the BCL2 inhibitor venetoclax (VEN) to an HMA results in a strong in vivo synergism [1]; however, outcome of some genetic risk groups is still unsatisfactory. Older patients with AML often carry* TP53* mutations [2, 3], associated with a very poor prognosis (ELN 2022) (https://www.leukemia-net.org/) [4]. Presently, *TP53*-mutant AML remains a formidable therapeutic challenge [5], urging the development of combination therapies for these patients.

In the DECIDER study (NCT00867672), the combination of DEC with the histone deacetylase inhibitor valproic acid (VPA) and/or all-*trans* retinoic acid (ATRA) was investigated in two hundred treatment-naive elderly patients with AML [6]. The trial showed a significant overall survival (OS) benefit with the addition of ATRA to DEC. In a post hoc analysis (*TP53* status being available for 168 of 200 patients), the 39 patients with a *TP53* mutation showed an overall response rate comparable to the 129 patients with *TP53* WT (ORR 23.1% vs. 15.5%) [7]. Among the 39 patients with *TP53* mutation (14 patients receiving DEC + ATRA and 25 DEC without ATRA), four had an OS of more than 2 years. One patient receiving DEC + ATRA who had a *TP53* mutation and a complex-monosomal karyotype lived for more than 3 years with continued dual treatment, despite the fact that such mutations are associated with a very poor prognosis. To better understand this counter-intuitive exceptional outcome, we performed an in-depth case study.

## Methods

The patient was enrolled in the DECIDER study in January 2014. This phase II study with a 2 × 2 factorial design is a prospective, randomized, observer blind, parallel group, multicenter study, with the primary endpoint being objective best overall response (complete remission [CR] and partial remission [PR]). Patients included were 60 years or older and unfit for standard induction therapy. The patients were randomized into four different treatment arms: (A) DEC alone, (B) DEC + VPA, (C) DEC + ATRA, and (D) DEC + VPA + ATRA.

For the *TP53* mutation analyses, bone marrow cells before treatment were used. The Illumina TruSight Myeloid Sequencing Panel was used for library preparation, an Illumina MiSeq device for sequencing. Whole-genome sequencing (WGS) was performed with TruSeq Nano Library Prep (Illumina) and NovaSeq 6000 (Illumina) Paired End 150 base pair sequencing.

The WGS data were processed using the nextflow workflow (https://github.com/CompEpigen/wf_WGS), where structural variants were called with manta (https://doi.org/10.1093/bioinformatics/btv710), copy-number alterations were detected using Control-FREEC (https://doi.org/10.1093/bioinformatics/btr670), the circos plot was made with figeno (https://doi.org/10.1093/bioinformatics/btae354), and mutations were called with mutect2 (https://doi.org/10.1101/861054). Since no matched normal sample was available, SNVs found in the gnomAD database (https://doi.org/10.1038/s41586-020-2308-7) were excluded. In addition, we used pysensembl to obtain the impact of the mutations at the protein level, and only considered non-synonymous mutations in 52 genes known to be mutated in AML: *DNMT3A, NPM1, FLT3, RUNX1, SF3B1, SRSF2, U2AF1, NF1, JAK2, TP53, IDH1, IDH2, NRAS, KRAS, KIT, TET1, TET2, CEBPA, WT1, PTPN11, ASXL1, ASXL2, EZH2, KMT2A, KMT2C, KMT2D, KMT2E, CREBBP, KDM6A, KAT6A, DNMT3B, NSD1, SUZ12, JARID2, ETV6, KDM3B, RB1, CEBPG, NCOR1, NCOR2, BCOR, GATA2, NOTCH1, NOTCH2, ZRSR2, PHF6, MED12, SMARCA2, SMARCA4, SMC1A, SMC3, and STAG2*.

## Results

This 82-year-old female patient was diagnosed with AML in January 2014. The patient was overall physically fit, ECOG performance status = 1 (Eastern Cooperative Oncology Group) despite her chronological age. She had no preceding hematological disorder and no comorbidities. She managed her daily living in an independent fashion (Activities of Daily Living [ADL] = 95%).

**Fig. 1:**
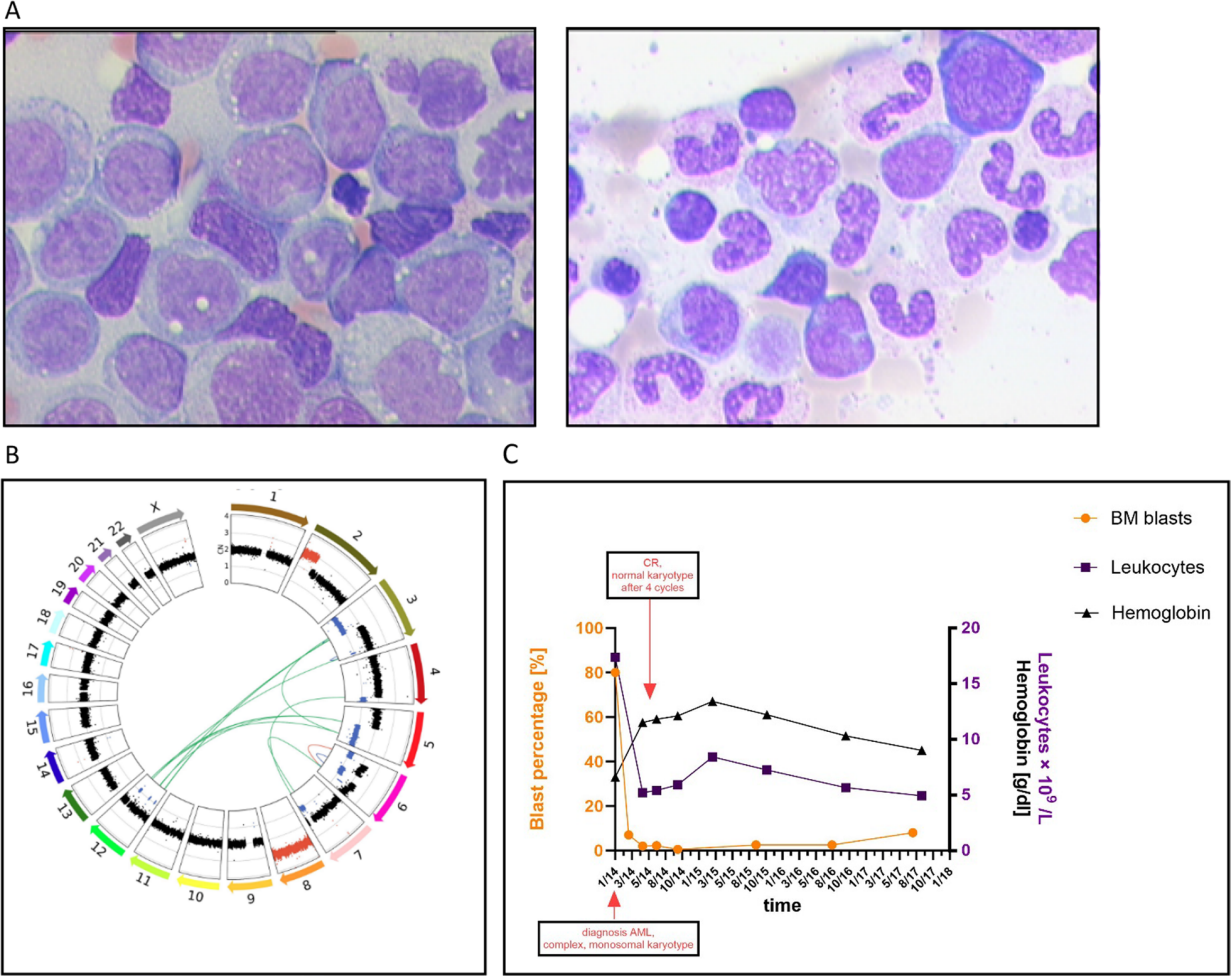
** A** Left panel: BM aspirate at diagnosis of AML (Jan. 2014) shows a dense blast infiltration, right panel: response to DEC + ATRA (October 2014); BM blast percentage 1%. **B** Circos plot showing copy-number alterations in BM cells obtained prior to treatment start, with losses in blue, gains in red, and diploid regions in black, and the structural variants (SVs) as green arcs observed in WGS data. **C** Schematic representation of clinical course; kinetics of Hb (black line), BM blast percentage in orange, and leukocyte kinetics in purple. Complex, monosomal karyotype at diagnosis (Jan. 2014), normal karyotype (July 2014); CR from May 2014 to July 2017 (8% BM blast). Therapy administered every 4 weeks (= one cycle), cycle 23–25, therapy administered without ATRA. Timeframe over 3.7 years from therapy initiation with DEC + ATRA until patient decided to stop treatment. Hb hemoglobin; DEC decitabine; ATRA all-trans retinoic acid; BM bone marrow; and CR complete remission

The diagnosis of an AML M2 (FAB) was made, with a complex-monosomal karyotype: 46,XX,-3,add (4)(q31),-5,-6,add(7)(q36), + 8,add(19)(q13), + mar, + r[20]/46,XX [2]. WGS revealed a complex karyotype including del(5q) and del(7q) (Fig. 1B), without chromothripsis, and no enhancer hijacking events were detected by pyjacker (GitHub—CompEpigen/pyjacker).

In line with this highly complex karyotype, the AML carried a non-disruptive, in-frame deletion mutation in* TP53* with a variant allele frequency (VAF) of 90% (p.Cys238_Asn239delinsTyr) (Table 1). This deletion led to the substitution of the two amino acids (cysteine and asparagine) by a single tyrosine. The mutation found in this patient lies within the DNA-binding domain of the p53 protein, in one of the mutation hotspot regions [8].

**Table 1:** Mutations detected by whole-genome sequencing

Affected gene	Chromosome	AA change	VAF (%)
*TP53*	17	p.Cys238_Asn239delinsTyr	90
*CSF3R*	1	p.Gln369Glu	57.7
*NF1*	17	p.Ala203Ser	47.3

AA, amino acid and VAF, variant allele frequency

In addition to the *TP53* mutation, two additional gene mutations, in *CSF3R* (p.Gln369Glu) with a VAF of 57.7%, and *NF1* (p.Ala203Ser) with a VAF of 47.2%, respectively, were found by WGS.

She was enrolled on the DECIDER study and was randomized into Arm C (DEC 20 mg/m^2^ d1–d5 and ATRA 45 mg/m^2^ d6–28). After 4 therapy cycles, the AML was in complete remission (CR) (Table 2) with 2 % BM blasts and cytogenetic conversion to normal karyotype (Fig. 1C).

**Table 2:** Laboratory data at initial diagnosis of AML (January 2014) and at complete remission to DEC + ATRA treatment (March 2015); blast percentage from October 2014 (see Fig. 1A, right panel)

Variable	Reference range	Initial diagnosis of AML	Complete remission
White blood cells, × 10^9^ /L	4.0–10.4	17.36	8.43
Absolute neutrophil count, × 10^9^ /L	1.9–7.3	4.34	5.48
Platelets, × 10^9^ /L	176–391	263	227
Red blood cells, × 10^12^ /L	3.9–5.2	2.6	4.6
Blasts in BM, %	0.3–5.0	80	1
Blasts in PB, %	–	13	0
Hb, g/dl	11.6–15.5	6.6	13.4

BM, bone marrow; PB, peripheral blood; and Hb, hemoglobin

The therapy was administered for a total of 30 cycles, with continued complete hematological remission. Eventually, she presented with a slowly evolving hematological relapse (8% bone marrow blasts), and at the end of the 30th cycle (3.7 years from AML diagnosis), the patient wished to stop treatment, despite only modest AML activity (i.e., no full-blown relapse), opting for home care instead.

## Discussion

In the DECIDER trial, the addition of ATRA to DEC led to a higher overall response rate (ORR) and significantly improved OS, even in patients with adverse cytogenetics. This was recently exemplified by a patient (sole del(5q), no mutations or deletions of *TP53*), carrying several high-risk features (secondary AML, adverse genetics, and older age), achieving a CR and an OS of 5.3 years [9]. The patient showed an above-average survival, receiving an exceptional number of 52 DEC + ATRA cycles with very good tolerance when adding ATRA to DEC, with no additional toxicities such as skin reactions [9].

In the DECIDER subgroup of 39 patients with *TP53* mutation, four had an OS of more than 2 years, two receiving the addition of ATRA. One of them, the 82-year-old patient reported here, received a total of 30 DEC + ATRA cycles. It is notable that this informative patient attained a robust cytogenetic remission after the 4th therapy cycle (Fig. 1C). Even when eventually she declined further treatment due to failing general health, no massive secondary resistance had developed. This is a remarkably long disease and treatment course, considering the presence of the *TP53* mutation with a VAF of 90% and the complex-monosomal karyotype. A comparison of allografted patients with lower *TP53* MUT VAF at the time of diagnosis with those with higher *TP53* VAF revealed that the former group exhibited superior outcomes [10].

*TP53* is the most commonly mutated gene in human cancers; in patients with AML, six hotspot mutations (R175H, G245S, R248Q/W, R249S, R273H/S, and R282W) within the DNA-binding domain are the most frequent [11]. Current standard therapies including intensive chemotherapy and allogeneic stem cell transplantation are often not effective in AML patients with *TP53* mutation [12]. Consequently, most AML patients with mutations in *TP53* succumb to leukemia within a few months. In the VIALE-A trial, for patients with AML ineligible to receive intensive chemotherapy, the combination of AZA plus VEN was an effective treatment regimen that led to significant improvements in CR rate and OS [1]. The specific *TP53* mutation found here (Fig. 1B) lies within one of the mutation hotspot regions [8] and was previously described in one case of esophageal cancer (Mutation (bmtongji.cn) ID 113). Another nucleotide change leading to p.Cys238Tyr reveals an EAp53 score of 92.66, with a high risk in head and neck cancer [13]. As often co-occurring with *TP53* mutations, losses of several autosomal chromosomes were observed. Notably, reactivation by HMAs of transposable elements (TEs) and other genes triggering an immune response occurs preferentially on monosomic chromosomes [14]; de-repression of TEs is enhanced by the addition of ATRA to DEC [15]; hence, it is tempting to speculate this cooperative effect may also boost the antileukemic activity in AML/MDS patients with monosomal karyotypes.

This patient reported here spent most of her time from the start of DEC + ATRA treatment at home, retaining her independence and autonomy, with stabilized quality of life and minimized “time toxicity” of unplanned hospitalizations. No ATRA-related toxicities were observed. The combination of DEC + ATRA could be an alternative for patients not tolerating DEC + VEN treatment. We showed activity of DEC + ATRA in *TP53* mutated AML cell lines, supporting further preclinical and clinical investigations of HMA and retinoid combination therapies [15]. In the currently recruiting DECIDER-2 phase III trial (AMLSG 32–21; EudraCT No. 2020-005495-36), the potential benefit of the addition of ATRA to DEC + VEN is studied in a placebo-controlled fashion.

In summary, this case describes an AML patient with a *TP53* mutation, indicating a very poor prognosis. Nevertheless, the patient showed a survival that exceeded all expectations with the combination treatment of 30 cycles of DEC + ATRA. The patient's quality of life remained stable until shortly before she chose to discontinue treatment. This case is significant because older patients with AML, even with the availability of HMA combination therapies, still have a poor prognosis if allografting is not possible. Therefore, stabilizing their quality of life is one of the primary treatment goals. Of course, the psychological resources of patients (motivation, compliance, resilience, etc.) are an important aspect of a therapy where long-term disease control can only be achieved if patients demonstrate discipline and autonomy throughout the treatment, as in this particular case, where successful outcome was achieved.

## Data Availability

The data that support the findings of this study are available on request from the corresponding author, [M.L.].
